# 4EGI-1 targets breast cancer stem cells by selective inhibition of translation that persists in CSC maintenance, proliferation and metastasis

**DOI:** 10.18632/oncotarget.2112

**Published:** 2014-06-18

**Authors:** Tingfang Yi, Eihab Kabha, Evangelos Papadopoulos, Gerhard Wagner

**Affiliations:** ^1^ Department of Biological Chemistry and Molecular Pharmacology, Harvard Medical School, Boston, MA

**Keywords:** cancer stem cells, eIF4E, translation, 4EGI-1

## Abstract

Cancer death is a leading cause of global mortality. An estimated 14.1 million new cancer cases and 8.2 million cancer deaths occurred worldwide in 2012 alone. Cancer stem cells (CSCs) within tumors are essential for tumor metastasis and reoccurrence, the key factors of cancer lethality. Here we report that 4EGI-1, an inhibitor of the interaction between translation initiation factors eIF4E1 and eIF4G1 effectively inhibits breast CSCs through selectively reducing translation persistent in breast CSCs. Translation initiation factor eIF4E1 is significantly enhanced in breast CSCs in comparison to non-CSC breast cancer cells. 4EGI-1 presents increased cytotoxicity to breast CSCs compared to non-CSC breast cancer cells. 4EGI-1 promotes breast CSC differentiation and represses breast CSC induced tube-like structure formation of human umbilical vein endothelial cells (HUVECs). 4EGI-1 isomers suppress breast CSC tumorangiogenesis and tumor growth in vivo. In addition, 4EGI-1 decreases proliferation in and induces apoptosis into breast CSC tumor cells. Furthermore, 4EGI-1 selectively inhibits translation of mRNAs encoding NANOG, OCT4, CXCR4, c-MYC and VEGF in breast CSC tumors. Our study demonstrated that 4EGI-1 targets breast CSCs through selective inhibition of translation critical for breast CSCs, suggesting that selective translation initiation interference might be an avenue targeting CSCs within tumors.

## INTRODUCTION

Cancer is a leading cause of mortality worldwide. Global cancer burden increased to 14.1 million new cases and 8.2 million cancer deaths in 2012 from 12.7 million and 7.6 million respectively, in 2008. Cancer metastasis and reoccurrence are the major resources of cancer lethality. Cancer stem cells (CSCs) form a subpopulation of cells within tumors and are essential for tumor dissemination and relapse[1]. The acquired properties that make CSCs adaptive to micro-environmental stresses and enhanced resistance to chemo-/radiation-therapy, present a challenge for targeting CSCs within tumors[2-4]. Heterogenetic CSCs are believed to acquire diverse adaptive changes in genetic, epigenetic, signal transduction, metabolic, transcriptional and translational levels[5, 6]. Translation is essential for all aspects of tumor evolution, including tumorigenesis, tumorangiogenesis, tumor growth, metastasis, CSC heterogeneity, and drug resistance[7, 8]. Protein synthesis is central for CSCs self-renewal, maintenance, differentiation, growth and dissemination.

Translation generally initiates with the recruitment of eIF4F to the 5'- m^7^GpppN cap of messenger RNA (mRNA)[9, 10]. eIF4F is a complex composed of the cap-binding protein eIF4E1, scaffold protein eIF4G1 and RNA helicase eIF4A. The eIF4E1/eIF4G1 interaction is necessary for eIF4F assembly[10-12]. The mRNAs encoding proteins important for cell proliferation and growth frequently harbor secondary structure and/or long 5'-UTR, or other regulatory elements. Translation of mRNAs with structured and/or long 5'-UTR is highly eIF4E1 dependent[13, 14]. Consistently, enhanced eIF4E1 levels and/or activities have been demonstrated in a number of cancers in clinical observations[15-18]. The human eIF4E family has three members of eIF4E1, eIF4E2 and eIF4E3. The eIF4E2-RBM4-HIF-2α complex can initiate translation but eIF4E2 does not bind to eIF4G1[19, 20]. eIF4E3 competes with eIF4E1 in binding cap and acts as a tumor suppressor[21]. Multiple proteins have been demonstrated to be enhanced in breast CSCs, such as the CSC markers NANOG and OCT4[22], the metastasis regulator CXCR4[23, 24], the epithelial–mesenchymal transition (EMT)[25] regulator c-MYB[26], or the cell proliferation key factors c-MYC[27] and cyclin D1[28, 29].

Due to the central roles in cancer evolution, translation is targeted in anti-cancer drug development. Several agents targeting translation initiation have been developed[30, 31], including the small molecule 4EGI-1[32]. 4EGI-1 binds eIF4E1, prevents recruitment of eIF4E1 to eIF4G1. The compound mimics the function of 4E-binding protein 1 (4E-BP1), which regulates eIF4E1-eIF4G interaction in a phosphorylation–dependent manner; however, 4EGI-1 does not displace 4E-BP1 from eIF4E1[32]. 4EGI-1 exhibits inhibitory effects in several cancer cell lines[33-35].

Recently, we have isolated and identified a small fast adhesion subpopulation (CD44^high^/CD24^low^)^FA^ from HMLER human mammary epithelial cancer cells [1, 2] that show the typical properties of cancer stem cells and are referred to as breast cancer stem cells (CSCs)[36]. These breast CSCs showed enhanced drug resistance to the anti-cancer drugs actinomycin D and camptothecin (about 10-fold) compared to non-CSCs [36]. However, it is unknown whether 4EGI-1 inhibits breast CSCs and suppresses breast CSC tumor growth, and whether 4EGI-1 interferes with translation selectively in breast CSCs is elusive.

We investigated the effects of 4EGI-1 on breast CSCs of HMLER (CD44^high^/CD24^low^)^FA^ cells *in vitro* and breast CSC tumorangiogenesis and tumor growth in xenografted tumor model *in vivo*. We found that eIF4E1 is significantly increased in breast CSCs comparing to non-CSC breast cancer cells. 4EGI-1 selectively inhibits breast CSCs in comparison to non-CSC breast cancer cells. Both 4EGI-1 isomers repress breast CSC tumorangiogenesis and tumor growth in mouse model. They inhibit cell proliferation and increase cell apoptosis in breast CSC tumors. 4EGI-1 selectively inhibits translation of mRNAs of *NANOG, OCT4, CXCR4, c-MYB, c-MYC* and *cyclin D1* in breast CSC tumors. These results suggest that 4EGI-1 targets breast CSCs through selectively inhibits translation of mRNAs essential for breast CSCs.

## RESULTS

### Translation initiation factor eIF4E1 is enhanced in breast CSCs

**Figure 1:**
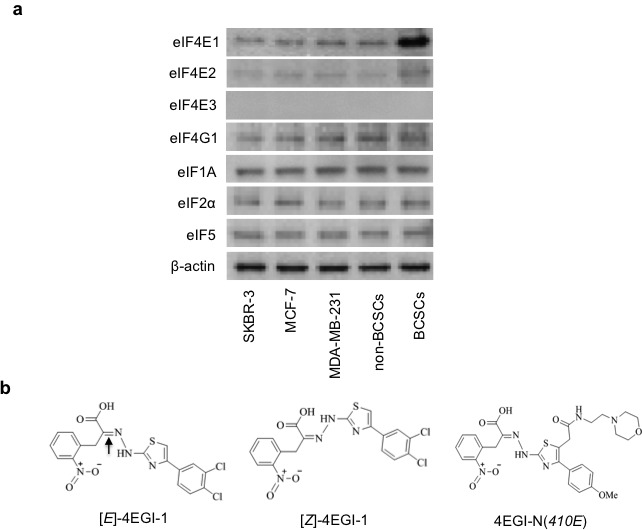
Translation initiation factor eIF4E1 is enhanced in breast CSCs and structures of compounds (a) Western blot analyses of translation initiation factor abundance in SKBR-3, MCF-7, MDA-MB-231 breast cancer cells, breast CSCs (BCSCs) of HMLER (CD44^high^/CD24^low^)^FA^ cells and non-BCSCs of HMLER (CD44^high^/CD24^low^)^SA^ breast cancer cells. β-actin was used as a loading control. (b) Structures of 4EGI-1 isomers ([*E*]- and [*Z*]-isomer) and inactive analogue 4EGI-N(*410E*). Arrow shows the double bonds.

To investigate the underlying translational mechanisms that are specifically required for CSC character maintenance of breast CSCs (BCSCs), we compared the cellular protein abundances of multiple translation initiation factors by Western blot assays in breast CSCs HMLER (CD44^high^/CD24^low^)^FA^ cells and multiple non-CSC breast cancer cells (non-BCSCs). We found that eIF4E1 (also named eIF4E), but not eIF4E2, eIF4G1, eIF1A, eIF2α, or eIF5, is significantly increased in BCSCs, in comparison to SKBR-3, MCF-7 and MDA-MB-231 breast cancer cells and non-BCSCs of HMLER (CD44^high^/CD24^low^)^SA^ cells (Fig.1A). eIF4E3 is hardly detectable in all these cancer cells. These results suggest that the activation of eIF4E-dependent translation is enhanced in these BCSCs.

### Selective inhibition of breast CSCs by 4EGI-1 comparing to non-CSCs

4EGI-1 consists of two isomers: [*E*]- and [*Z*]-4EGI-1. To evaluate the effects of 4EGI-1, we synthesized and identified an inactive analogue 4EGI-N(*410E*) (Fig.1B). We compared the inhibitory effects of 4EGI-1 isomers on breast CSCs and multiple non-CSC breast cancer cells by ATP concentration based cell viability assays. We observed that the IC_50_ of 4EGI-1 is about 30μM on SKBR-3, MCF-7 and MDA-MB-231 breast cancer cells, about 22μM on non-CSCs, and about 11μM ([*E*]-)/10μM ([*Z*]-) on breast CSCs (Table 1). The above data indicate that 4EGI-1 presents increased cytotoxicity (>2-fold) to breast CSCs comparing to non-CSC breast cancer cells, which is in agreement with the significantly enhanced eIF4E1 in breast CSCs (Fig. 1A).

**Table 1:** Cytotoxicity of 4EGI-1 isomers on breast CSCs and breast cancer cells

BCSCs and breast cancer cells	IC_50_ of [E]-4EGI-1(μM)	IC_50_ of [Z]-4EGI-1(μM)
SKBR-3	30±1.5	29±1.3
MCF-7	30±0.9	30±1.1
MDA-MB-231	32±1.2	30±1.4
Non-BCSCs of HMLER(CD44^high^/CD24^low^)^SA^	24±1.1	22±0.7
BCSCs of HMLER(CD44^high^/CD24^low^)^FA^	11±0.8	10±0.6

Note: IC_50_ means inhibitory concentration that leads to 50% cell viability decrease.

### 4EGI-1 promotes breast CSC differentiation

**Figure 2:**
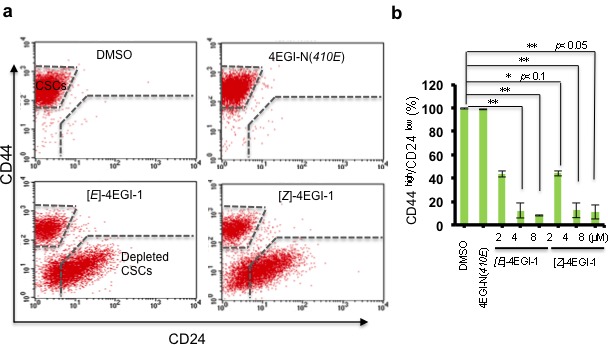
4EGI-1 promotes breast CSC differentiation (a) Representative fluorescence-activated cell sorting (FACS) images. CD44^high^/CD24^low^ population cells and CD44^low^/CD24^high^ population cells with DMSO, 4μM 4EGI-N(*410E*), 4μM[*E*]-4EGI-1, or 4μM[*Z*]-4EGI-1 treatment for three days were shown. Both 4EGI-1 isomers promote breast CSC differentiation. (b) Statistical analyses of 4EGI-1 effects on CD44^high^/CD24^low^ population cell percentage of breast CSCs. Average CD44^high^/CD24^low^ percentages were shown (mean ± SD, *t*-test, two-tailed). *: *p*< 0.1; **: *p*< 0.05; ***: *p*< 0.01.

CSC maintenance and differentiation are important for CSC tumorigenesis and tumor evolution[37]. CD44^high^/CD24^low^ is one of the defining characteristics of these breast CSCs, whereas CD44^low^/CD24^high^ represents the CSC-depleted population of these breast cancer cells. We treated the breast CSCs with 4EGI-1 isomers, an inactivate analogue 4EGI-N(*410E*) and vehicle with series of concentrations for three days followed by flow cytometry analyses with CD44 and CD24 antibodies. 4EGI-1 isomers significantly decreased the percentage of CD44^high^/CD24^low^ population cells and increased the population of CD44^low^/CD24^high^ cells (Fig. 2A-2B). 4μM [*E*]- or [*Z*]-4EGI-1 depleted most of breast CSCs. Importantly, inactive analogue 4EGI-N(*410E*) did not affect breast CSCs under the same conditions (Fig. 2A-2B). These results suggested that 4EGI-1 potently depletes breast CSCs and promotes breast CSC differentiation.

### 4EGI-1 inhibits breast CSC induced HUVEC tube-like structure formation

**Figure 3:**
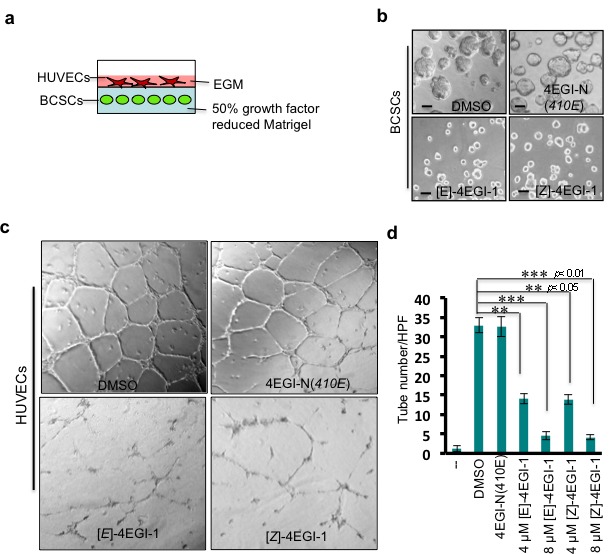
4EGI-1inhibits breast CSC induced HUVEC tube-like structure formation (a) Diagram of HUVEC-breast CSC sandwich. Breast CSCs were embedded in 50% growth factor reduced Matrigel (mixed with 50% MEGM medium). Human umbilical vein endothelial cells (HUVECs) were planted on Matrigel mixture in endothelial growth medium (EGM). (b) Representative images of breast CSC mammospheres (top panels) or breast CSCs (bottom panels) in Matrigel mixture. Breast CSCs were pretreated with DMSO, 8μM 4EGI-N(*410E*), 8μM[*E*]-4EGI-1, or 8μM[*Z*]-4EGI-1 for 1 day, followed by culturing in Matrigel mixture for three days. Images were taken at the third day in Matrigel mixture. Bar=10μm. (c) Representative images of HUVEC tube-like structures. HUVECs were cultured on Matrigel mixture containing 8μM compound pretreated breast CSCs for 24hr. Images were taken at 24hr. Breast CSCs (mammospheres) promote HUVEC tube formation, and 4EGI-1 inhibits this capacity of breast CSCs. (d) Statistical analyses of 4EGI-1 effects on 4EGI-1 effects on breast CSC stimulation of HUVEC tube formation. “_ _” means Matrigel mixture contains no breast CSCs. 50% growth factor reduced Matrigel does not lead to HUVEC tube formation. Average tube numbers per high performance field (20×) were shown (mean ± SD, *t*-test, two-tailed). **: *p*< 0.05; ***: *p*< 0.01.

Angiogenesis is the process of new vessel generation and growth, which is important for tumor growth and metastasis[38]. CSCs produce multiple proangiogenic factors to stimulate tumor angiogenesis[39]. To examine whether 4EGI-1 inhibits proangiogenic factor protein synthesis in breast CSCs, we employed human umbilical vein endothelial cells (HUVECs). We pretreated breast CSCs with compounds at the indicated concentrations for 3 hours. Then, we embedded these breast CSCs in 50% growth factor reduced Matrigel (mixed with 50% breast CSC medium MEGM) and planted HUVECs on the surface of these Matrigel mixtures with endothelial cell growth medium (EGM) (Fig. 3A). After incubation at 37°C for two days, breast CSCs pretreated with DMSO and inactivate analogue 4EGI-N(*410E*) grew into tumorspheres, while those treated with 4EGI-1 isomers did not (Fig. 3B). HUVECs cultured on the surface of Matrigel mixture with DMSO pretreated breast CSCs formed tube-like structures (33±2 tubes/HPF, high performance field, 20×), and 32.6±2.58 tubes/HPF with 4EGI-N(*410E*) pretreated breast CSCs. In contrast, HUVECs only formed 4.5±1 tubes/HPF and 4.1±0.7 tubes/HPF with 8μM [*E*]- and [*Z*]-isomer pretreated breast CSCs, respectively (Fig. 3C-D). Importantly, 4EGI-N(*410E*) did not affect HUVEC tube formation and HUVECs hardly form tubes on growth factor reduced Matrigel (Fig. 3D). These results demonstrated that 4EGI-1 inhibits proangiogenic factor production in breast CSCs and significantly represses breast CSC promoted HUVEC tube formation.

### 4EGI-1 suppresses breast CSC tumor growth and tumorangiogenesis

**Figure 4:**
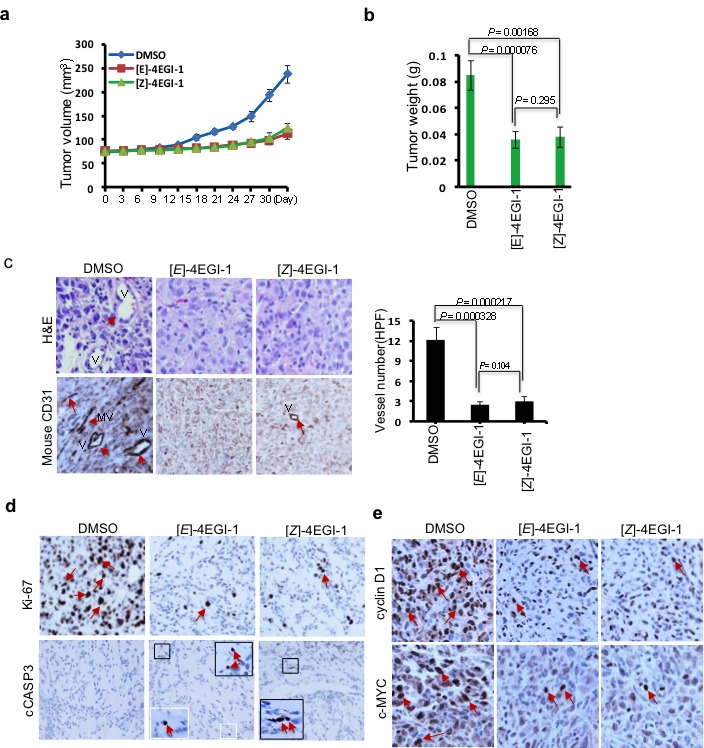
4EGI-1 suppresses breast CSC tumor growth and tumorangiogenesis *in vivo* (a) The graph represents average tumor volumes of each group over time. The average tumor volume from DMSO treated group increased from 74.30±2.43 to 237.4± 18.6mm^3^, whereas that from [*E*]-4EGI-1 treated group increased from 76.87±1.96 to 113.2±11.4 mm^3^, and that from [*Z*]-4EGI-1 increased from 75.01±1.75 to 123.9 ±10.8 mm^3^. Bar represents tumor volume mean ± SD (n=5 mice, *t*-test, *p*<0.001). (b) Average tumor weights of each group. The average tumor weight from DMSO treated group is 0.08524±0.01145g, whereas that from [*E*]-4EGI-1 treated group is 0.036± 0.00618g and that from [*Z*]-4EGI-1 treated group is 0.038±0.0077g (mean ± SD, n=5, *t*-test). (c) Representative H&E staining (top panels) and immunohistostaining (bottom panels) images of anti-mouse CD31 antibody in tumor cross-sections. Left panel: Statistical data from immunohistostaining analyses expressed as average tumor vessel number per high performance field (HPF, 200×) with 12.2±1.92 vessel/HPF in DMSO treated tumor group, 2.4±0.54 vessel/HPF in [*E*]-4EGI-1treated tumor group, and 3±0.7 vessel/HPF in [*E*]-4EGI-1treated tumor group (mean ± SD, n=6, *t*-test). Arrows indicate vessels and microvessels. V: vessels; MV: microvessels. (d) Representative immunohistostaining images of tumor cross-sections with anti-Ki-67 and anti-cleaved Caspase 3 (cCASP3) antibodies. Arrows indicate signals of Ki-67 or cCASP3. Framed areas show the enlarged images of cCASP3 signals. (e) Representative immunohistostaining images of cross-sections with anti-cyclin D1 and anti-c-MYC antibodies. Arrows indicate signals of cyclin D1 or c-MYC.

To investigate the effects of 4EGI-1 on breast CSC tumor growth *in vivo*, we employed xenografted tumor model in mouse. After the breast CSC tumor formation (tumor volume is about 75 mm^3^), we injected both 4EGI-1 isomers 75 mg/kg/day (5 mice for each group) by intraperitoneal injection (*I.P.)* for 30 days. Tumor volumes were measured every three days. At the 30^th^ day, the average breast CSC tumor volume of vehicle treatment was 237.4±18.6 mm^3^, whereas the average breast CSC tumor volumes of [*E*]- and [*Z*]- isomer treated were 113.2±11.4 mm^3^ and 123.9±10.8 mm^3^, respectively (Fig. 4A). The average breast CSC tumor weights of vehicle, [*E*]-, [*Z*]- isomer treated were 0.0825±0.01145g, 0.036±0.00618g, 0.038±0.0077g, respectively (Fig. 4B). These data demonstrated that 4EGI-1 effectively suppressed breast CSC tumor growth *in vivo*. Then, we performed hematoxylin and eosin (H&E) staining and immunohistostaining with anti-mouse CD31 (vascular endothelial cell marker) antibodies with breast CSC tumor sections. 4EGI-1 significantly decreased vessel numbers in breast CSC tumors (Fig. 4C), suggesting that 4EGI-1 inhibits angiogenesis in breast CSC tumors *in vivo*. Next, we performed immunohistostaining to detect cell proliferation marker Ki-67 and cell apoptosis marker cleaved CASP3 (cCASP3) in these breast CSC tumor sections. 4EGI-1 decreased Ki-67 in most breast CSC tumor cells and increased cCASP3 in a few breast CSC tumor cells (Fig. 4D), suggesting that 4EGI-1 significantly inhibits breast CSC proliferation and can induce apoptosis in breast CSCs *in vivo*. Furthermore, we found 4EGI-1 strikingly decreased cell proliferation key regulators cyclin D1 and c-MYC in breast CSC tumor cells (Fig. 4E), indicating that 4EGI-1 inhibits translation essential for cell proliferation in breast CSCs.

### 4EGI-1 selectively inhibits translation that persists in CSC maintenance and dissemination

**Figure 5:**
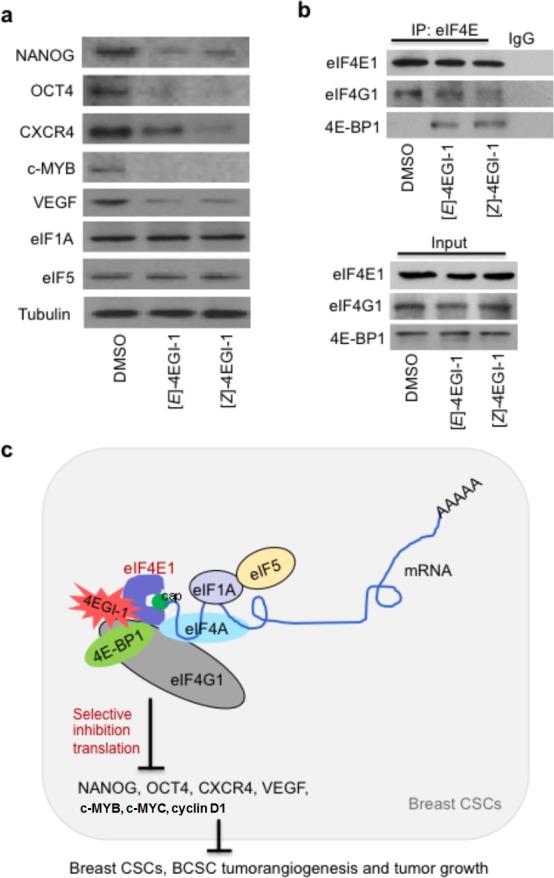
4EGI-1 selectively inhibits translation in breast CSC tumors (a) 4EGI-1 decreases NANOG, OCT4, CXCR4, c-MYB and VEGF, but not eIF1A or eIF5, in breast CSC tumors. Western blot assays of 4EGI-1 effects on translation in breast CSC tumors. Total proteins were extracted from tumor tissues. Western blot were performed three independent experiments with indicated antibodies. Tubulin was used as a loading control. (b) 4EGI-1 disrupts eIF4E1-eIF4G1 interaction and increases stability of 4E-BP1 binding to eIF4E1 in breast CSC tumors. Co-immunoprecipitation assays were performed using anti-eIF4E antibody or control IgG. “Input” represents 10% of total protein used in immunoprecipitation. (c) Schematic diagram for enhanced eIF4E1 in breast CSCs, selective translation initiation interference by 4EGI-1 in breast CSCs, and inhibitory effects of 4EGI-1 on breast CSC tumorangiogenesis and tumor growth. 4EGI-1 binds to eIF4E1, inhibits eIF4E1-eIF4G1 interaction, and increase 4E-BP1 binding to eIF4E1. 4EGI-1 selectively inhibits translation essential for breast CSC maintenance, proliferation and metastasis, and suppresses breast CSC tumorangiogenesis and tumor growth.

To examine the 4EGI-1 effects on translation essential for CSC maintenance and dissemination, we performed Western blot with cellular proteins extracted from these breast CSC tumors. 4EGI-1 treated tumor cells presented decreased CSC pluripotency markers NANOG and OCT4, tumor metastasis regulator CXCR4, EMT mediator c-MYB and proangiogenic factor VEGF[40, 41], but not translation initiation factors eIF1A or eIF5, comparing to vehicle treated breast CSC tumor cells *in vivo* (Fig. 5A). Previously, we reported that 4EGI-1 inhibits translation of several oncogene mRNAs without affecting their transcription in multiple cancer cell lines at 24hr under normoxia and at 12hr under hypoxia conditions *in vitro*[16, 32]. These observations suggested that the indicated decreased protein levels in 4EGI-1 treated BCSC tumors were primarily, if not all, caused by selective inhibition of translation of mRNAs encoding these proteins. Importantly, we found that 4EGI-1 isomers inhibit the eIF4E1-eIF4G1 interaction and increase 4E-BP1 stability of binding to eIF4E1 in breast CSC tumor cells in vivo (Fig. 5B). These results demonstrated that 4EGI-1 selectively inhibits translation of mRNAs encoding proteins that persists in CSC maintenance, pro-angiogenesis and metastasis.

## DISCUSSION

Cancer stem cells are critical for tumor metastasis and reoccurrence, which are the major resources of cancer-related lethality. CSCs have acquired enhanced drug resistance. Here we found that small molecule 4EGI-1 preferentially inhibits breast CSCs compared to non-CSC breast cancer cells *in vitro* and effectively suppresses breast CSC tumor growth and tumorangiogenesis *in vivo*. 4EGI-1 promotes breast CSC differentiation, inhibits breast CSC tumorsphere growth and suppresses HUVEC tube formation. 4EGI-1 significantly inhibits breast CSC proliferation and can induce apoptosis in breast CSC tumor cells. Furthermore, we found that 4EGI-1 selectively inhibits translation of mRNAs encoding proteins that are enhanced in CSC maintenance, proliferation and metastasis, such as NANOG, OCT4, c-MYC, cyclin D1, CXCR4, VEGF and c-MYB, in breast CSC tumor cells. Our study suggests that selective translation initiation interference by 4EGI-1 inhibits breast CSC tumor growth and tumorangiogenesis (Fig. 5C).

Malignant cancer cells typically undergo extensive adaptive genetic, epigenetic and metabolic changes to support the heterogeneous malignant phenotypes, yet cell survival and proliferation may dependent on one or a subset of driver mutations[42, 43]. This continued dependence on the driver mutation is deferred to oncogene addiction[42]. Inhibition of the addictive oncoproteins can lead to therapeutic intervention, which is the basis for a considerable promise of current chemotherapy. However, the followed tumor reoccurrence, tumor metastasis and drug resistance suggest limits of using inhibitors targeting one or a few addictive oncoproteins. Although it is known that: 1) CSC quiescence decreases drug sensitivity; and 2) CSCs may never acquire addiction to the oncogene that drives disease development, the mechanisms of enhanced drug resistance of CSCs are elusive[4]. Despite of the extreme complexity of increased drug resistance of CSCs[45], it is possible to target the CSC-specific adaptive translational mechanisms that constantly produce proteins to support CSC maintenance, self-renewal and metastasis. Here, we found that translation initiation factor eIF4E1, but not eIF4E2, eIF4G1, eIF1A, eIF2α, or eIF5, is significantly enhanced in breast CSCs in comparison to non-CSC breast cancer cells. We show that the eIF4E1-eIF4G1 interaction inhibitor 4EGI-1 preferentially inhibits breast CSCs compared to non-CSC breast cancer cells, effectively suppresses breast CSC tumor growth, selectively inhibiting translation of mRNAs encoding NANOG, OCT4, CXCR4, c-MYC, cyclin D1, c-MYB and VEGF, but not eIF1A or eIF5. CXCR4 and c-MYC are addictive oncoproteins of many malignant tumors. Therefore, 4EGI-1 both inhibits addictive oncoproteins and interferes with the CSC adaptive protein synthesis machinery in breast CSCs. These results not only demonstrate that 4EGI-1 is a potential candidate for CSC-targeted cancer therapy, but more importantly, suggest that the eIF4E1-eIF4G1 interaction is an available target for the selective inhibition of CSCs.

It has been demonstrated that translation of mRNAs encoding proteins important for cell proliferation and survival is highly eIF4E1 dependent [15, 44]. Here we show that 4EGI-1 selectively inhibits translation essential for breast CSCs, including *NANOG, OCT4, CXCR4, c-MYC, c-MYB* and *VEGF*. Data shown here and elsewhere[32] have consistently evidenced that 4EGI-1 performs selective translation interference in breast CSCs and multiple cancer cell lines. Here we show that 4EGI-1 increases cytotoxicity to breast CSCs that exhibit enhanced drug resistance to anti-cancer drugs of Actinomycin D and Camptothecin (about 10-fold). This suggests that 4EGI-1 can selectively target breast CSCs. The intraperitoneal injection and suppression of breast CSC tumor growth, and the low side effects on mice (data not shown) in this study reflect the selective breast CSC inhibition by 4EGI-1. In short, 4EGI-1 exhibits capacities of both selective translation initiation interference and selective breast CSC inhibition.

CSC maintenance facilitates tumorigenesis and tumor reoccurrence[22], and CSC quiescence decreases sensitivity to inhibitors[45, 46]. Furthermore, CSC depletion reduces CSC tumorigenesis capacity. We found that 4EGI-1 inhibits breast CSC maintenance and promotes breast CSC differentiation with decreased CD44^high^/CD24^low^ population and increased CD44^low^/CD24^high^ population cells. Furthermore, 4EGI-1 decreased CSC markers of NANOG and OCT4 in breast CSC tumors, which associate with hypoxia (low oxygen) within solid tumors. These consistent results demonstrate that 4EGI-1 effectively promotes breast CSC differentiation by selectively impairing translation required for CSC maintenance.

Cancer cells have acquired capacities of uncontrolled proliferation and apoptosis evasion[47]. We found that 4EGI-1 significantly decreases cell proliferation, and c-MYC and cyclin D1 protein levels in breast CSC tumors. 4EGI-1 can induce apoptosis in CSCs within breast CSC tumors. These observations evidenced that selective translation initiation interference may be an avenue to effectively suppress proliferation in and induce apoptosis into CSCs. On the other hand, targeting CSC adaptive translation machinery may overcome: 1) inherent resistance to drugs targeting one or a few oncoproteins, which is caused by CSC heterogeneity and/or compensation of diverse/redundant oncoproteins in a type of CSCs[41]; and 2) acquired resistance due to mutations after drug treatment[48, 49].

We present here the first small molecule activities against breast cancer stem cells through selective translation initiation interference. Selective inhibition of translation of mRNAs encoding proteins essential for CSC maintenance, self-renewal, proliferation, differentiation, and dissemination could be an avenue to restrict and inhibit CSC activities, including CSC tumorangiogenesis and tumor growth. Targeting selective translation initiation is expected to overcome resistance due to mutations acquired during drug treatment and the related inherent CSC heterogeneity.

## MATERIAL AND METHODS

### Cells, antibodies, reagents and mice

HMLER cell line was kindly provided by Robert Weinberg (Whitehead Institute for Biomedical Research of Massachusetts Institute of Technology). HMLER(CD44^high^/ CD24^low^)^FA^ cells were prepared as previously described[36]. MEGM and MEBM media, EGM media, and Human Umbilical Vein Endothelial Cells (HUVECs) were purchased from Lonza. The [*E*]- and [*Z*]-4EGI-1were either self-synthesized or ordered from Speed Chemical. 4EGI-N (*410E*) was self-synthesized. All compounds were dissolved in DMSO. *NOD-SCID* (strain name: *NOD.CB17-Prkdcscid/J*) mice were ordered from The Jackson Laboratory. Anti-Ki-67(SP6) antibody was ordered from Vector (^#^VP-RM04). Anti-cleaved CASP3 (5A1E) antibody for immunostaining was ordered from Cell Signaling (^#^9664s). Anti-CD31 (mouse) antibody was ordered from Dako (^#^N1596). Anti-c-Myc (N-term) antibody used for immunostaining was ordered from Epitomics (^#^1472-1). Anti-cyclin D1 antibody used for immunostaining was ordered from Neomarkers (^#^RM-9104-S). Anti-eIF4E1, anti-eIF4G1, anti-4E-BP1, and anti-β-actin antibodies were ordered from Cell Signaling. Cancer cell lines of SKBR-3, MCF-7, MDA-MB-231 were ordered from ATCC. Anti-c-MYC and anti-cyclin D1 antibodies were ordered from Cell Signaling.

### Western blot assay

Cellular protein extraction and Western blot assays were performed as previously described with RIPA buffer (50mM Tris-HCl *p*H 7.4, 150mM NaCl, 1% Triton-X100, 0.1% SDS, 0.25% Na-deoxycholate, 1mM PMSF, 1×Roche complete mini protease inhibitor cocktail). Immuno blots were performed with indicated antibodies. All Western blot experiments were performed three times.

### Cell viability assay

1 × 10^4^ breast CSCs HMLER (CD44^high^/CD24^low^)^FA^ cells and other indicated breast cancer cells were treated with DMSO, or [*E*]-4EGI-1 or [*Z*]-4EGI-1 at series of concentrations for 24 hours. The cells were performed cell viability assays with CellTiter-Glo^®^ luminescent cell viability assay kit (Promega) according to the manual description. Three independent experiments were performed. Average IC_50_ results were shown (mean ± SD, *t*-test, two-tailed).

### Fluorescence-activated cell sorting (FACS)

Breast CSCs were treated with the indicated compounds at indicated concentrations for three days, followed by fluorescence-activated cell sorting (FACS) assay with FITC-conjugated anti-CD44 (Biosciences, G44-26) antibodies and PE-conjugated anti-CD24 (Biosciences, ML15) antibodies.

### HUVEC tube-like structure formation

A 200μl 50% growth factor reduced Matrigel mixture (BD Medical, mixed with MEGM at ratio of 1:1) was added to each well of 24-well plates followed by incubation at 37°C for 30 minutes. Breast CSCs were treated with DMSO, 4EGI-N(*410E*), [*E*] and [*Z*] at indicated concentrations for one day, and cultured on Matrigel mixture, and covered by another 150 μl Matrigel mixture followed by incubation at 37°C for 30 minutes. Then 0.3 ml MEGM was added to each well and the plates were incubated at 37°C, 5% CO_2_ for 3 days. Images of breast CSCs or tumorspheres were taken by Nikon camera. After aspiration of the MEGM medium, 4×10^4^ HUVECs were cultured into each well together with 0.3 ml EGM medium followed by incubation at 37°C, 5% CO_2_ for 24 hours. Images of HUVEC tube-like structure were taken by Nikon camera. The HUVEC tube-like structures in high performance fields (20×) were counted. The experiments were performed three independent times.

### Tumor xenografted assay

In the tumor xenografted assay, 1×10^5^ breast CSCs were mixed with 100μl Matrigel/DMEM mixture (Matrigel: DMEM = 1:2) (BD Bioscience). Breast CSCs/Matrigel/DMEM mixtures were injected into *NOD/SCID* female mice (the Jackson Laboratory) mammary glands by subcutaneous injection. After the tumor formation (about 75 mm^3^ in volume, 5 mice/group), DMSO, or 75mg/kg [*E*]-4EGI-1, or 75mg/kg [*Z*]-4EGI-1 was injected into the mice by intraperitoneal injection daily for 30 days. Tumor volumes were measured every three days. At the 30^th^ day, mice were sacrificed and tumors were excised. Tumors weights were measured. Tumor tissue samples were used for immunohistostaining, Western blot and immunoprecipitation analyses. The mouse experiments were performed according to the policies of Harvard Medical Animal Committee.

### Statistical analysis

Quantitative data were statistically analyzed (mean ± SD, *t*-test, two-tailed). Statistical significance was determined by *t*-test. Significance was expressed as: *: *p*< 0.1; **: *p*< 0.05; ***: *p*< 0.01, or with the *p*-value. *P*<0.05 was considered significant[2].
